# Squamoid eccrine ductal carcinoma*

**DOI:** 10.1590/abd1806-4841.20164682

**Published:** 2016

**Authors:** Maria Isabel Ramos Saraiva, Marcella Amaral Horta Barbosa Vieira, Larissa Karine Leite Portocarrero, Rafael Cavanellas Fraga, Priscila Kakizaki, Neusa Yuriko Sakai Valente

**Affiliations:** 1 Universidade de São Paulo (USP) – São Paulo (SP), Brazil; 2 Private Clinic – Juiz de Fora (MG), Brazil; 3 Private Clinic – Vitória (ES), Brazil; 4 Hospital do Servidor Público Estadual (HSPE) de São Paulo – São Paulo (SP), Brazil

**Keywords:** Carcinoma, Carcinoma, Skin Appendage, Head and Neck Neoplasms

## Abstract

Squamoid eccrine ductal carcinoma is an eccrine carcinoma subtype, and only twelve cases have been reported until now. It is a rare tumor and its histopathological diagnosis is difficult. Almost half of patients are misdiagnosed as squamous cell carcinoma by the incisional biopsy. We report the thirteenth case of squamoid eccrine ductal carcinoma. Female patient, 72 years old, in the last 6 months presenting erythematous, keratotic and ulcerated papules on the nose. The incisional biopsy diagnosed squamoid eccrine ductal carcinoma. After excision, histopathology revealed positive margins. A wideningmargins surgery and grafting were performed, which again resulted in positive margins. The patient was then referred for radiotherapy. After 25 sessions, the injury reappeared. After another surgery, although the intraoperative biopsy showed free surgical margins, the product of resection revealed persistent lesion. Distinction between squamoid eccrine ductal carcinoma and squamous cell carcinoma is important because of the more aggressive nature of the first, which requires wider margins surgery to avoid recurrence.

## INTRODUCTION

Squamoid eccrine ductal carcinoma (SEDC) is an extremely rare subtype of eccrine carcinoma (EC): only twelve cases were reported in the literature to date (Table 1).^1^ Due to the rarity of this tumor and to the difficulty of its histopathologic diagnosis, almost half of the cases are incorrectly diagnosed as squamous cell carcinoma (SCC) in the initial biopsy.^2^ The origin of lesion is controversial, as it may represent a SCC emerging from eccrine duct, an EC subtype with extensive squamous differentiation or a biphenotypic carcinoma.^1^ We report the thirteenth case of SEDC in the literature, whose diagnosis was possible in the incisional biopsy.

**Table 1:** Published papers describing cases of squamoid eccrine ductal carcinoma

Reference	Case	Gender	Age	Site	Treatment	Recurrence	Follow-up
Wong et al.^6^	1	M	81	Ear	Conventional excision	Yes	36 months^a^
"	2	F	85	Hand	Conventional excision	Not informed	Lost to follow-up
"	3	F	86	Armpit	Conventional excision	Not informed	Lost to follow-up
Herrero et al.^9^	4	M	41	Knee	Not informed	Not informed	Not informed
Kim et al.^1^	5	F	30	Neck	Mohs micrographic surgery	No	14 months
Chhibber et al.^5^	6	M	90	Forearm	Conventional excision	No	5 months
Kavand and	7	F	61	Big toe	Amputation	No	8 months
Cassarino^10^							
Terushkin et al.^3^	8	M	63	Malar region	Mohs micrographic surgery	No	10 months
Pusiol et al.^11^	9	F	54	Leg	Conventional excision	No	18 months
Jung et al.^4^	10	M	53	Occipital region	Conventional excision	Yes	5 months^b^
Clark et al.^12^	11	M	75	Clavicular region	Mohs micrographic surgery	No	12 months
Wang et al.^13^	12	F	91	Chirodactyl	Amputation	No	2 months^c^
Current case	13	F	72	Nose	Conventional excision	No	23 months

a Three recurrences despite complete surgical excision ;

b With lymph node involvement;

c With metastasis

## CASE REPORT

**Figure 1:**
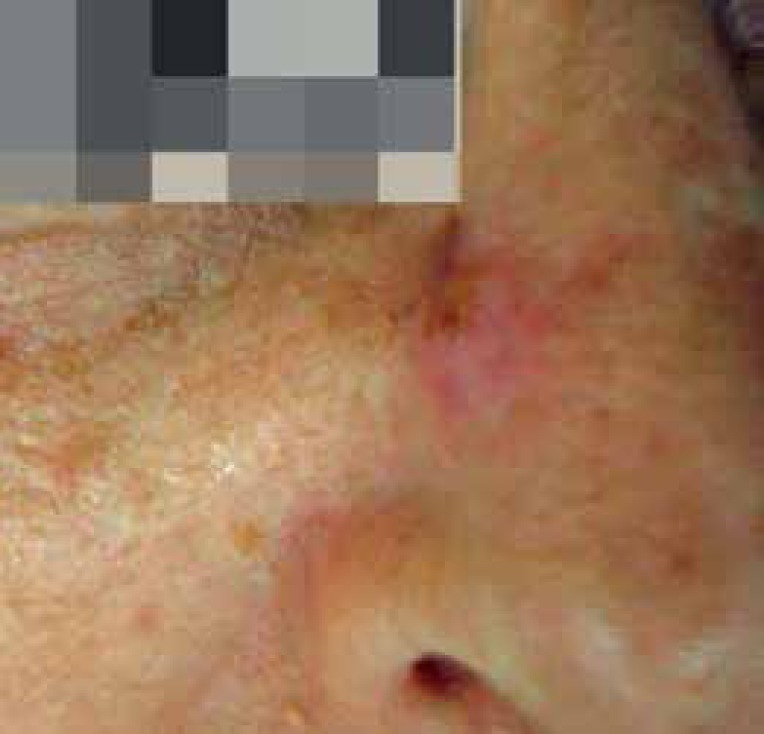
Clinical aspect of the lesion: erythematous papule, slightly keratotic and ulcerated in the nasal dorsum at the right

**Figure 2:**
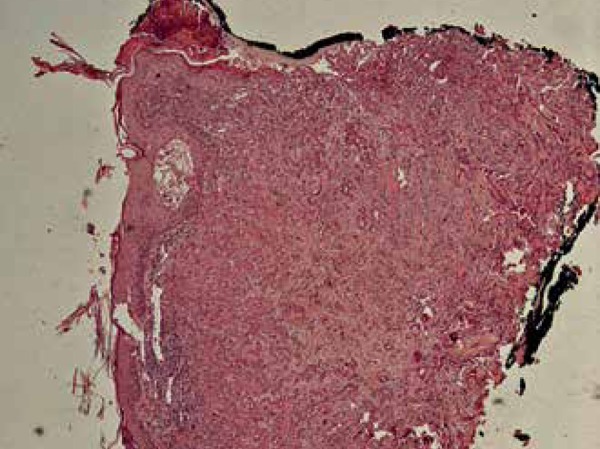
Histopathology revealed eccrine ductal carcinoma with squamous differentiation

**Figure 3:**
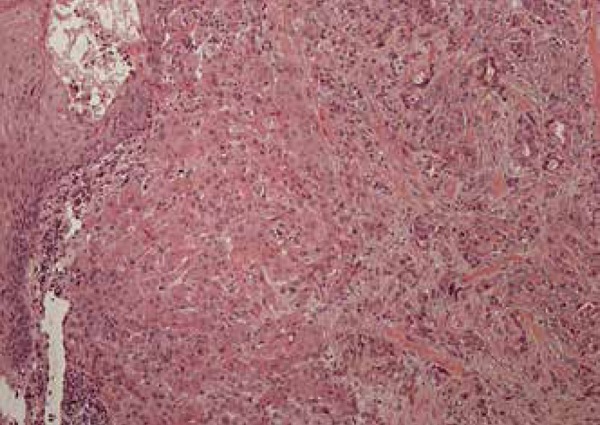
Eccrine ductal carcinoma with squamous differentiation in higher increase

**Figure 4:**
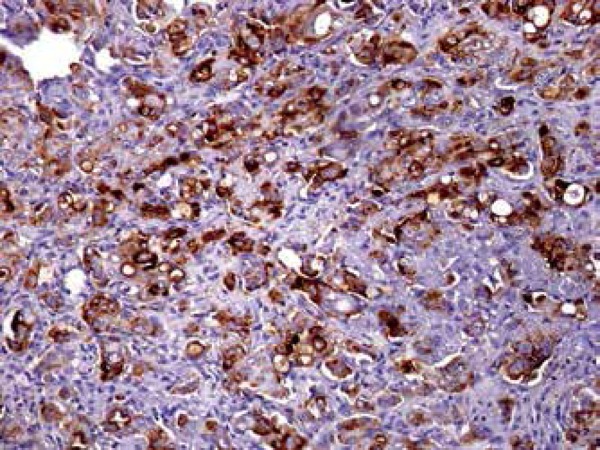
Immunohistochemistry reveals positivity for EMA

**Figure 5:**
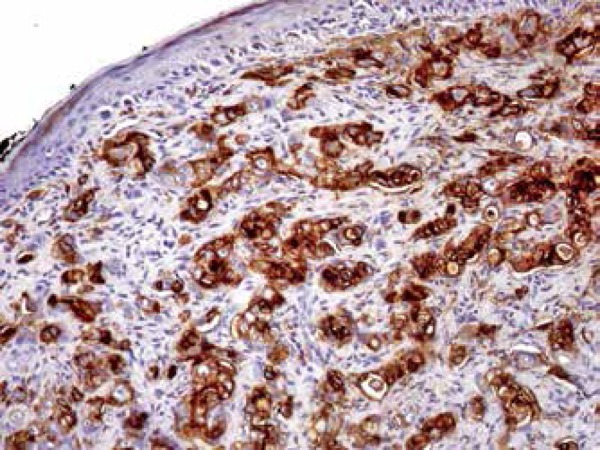
Immunohistochemistry reveals positivity for CEA

Female patient, 72 years, presenting erythematous papule for the last six months, slightly keratotic and ulcerated in the nasal dorsum to the right (Figure 1). As comorbidities, she had systemic hypertension, dyslipidemia and dyspepsia, for which she was in use of captopril, hydrochlorothiazide, simvastatin and omeprazole. An incisional biopsy of the lesion was performed, and the histological diagnosis was SEDC, corroborated by immunohistochemistry: epithelial membrane antigen (EMA) and carcinoembryonic antigen (CEA) were positive; cytokeratin 7 (CK7) was negative (Figures 2 to 5). The patient underwent surgical excision of the lesion twice, and histopathological examinations revealed, in both times, positive margins and infiltration of the hypodermis and of the striated muscle. We opted for adjuvant radiotherapy in the ala of the nose with curative purpose, in linear accelerator with energy beam of 6 mV at a dose of 66 Gy (33 fractions of cGy per cycle) for two months. Five months later, the patient presented reappearance of the lesion, whose biopsy demonstrated recurrence of cancer, and surgical treatment was indicated again. The intraoperative frozen section biopsy showed lateral and deep margins and septal cartilage free of neoplastic involvement. Nasal reconstruction was made with paramedian flap and cartilage grafting of the right ear shell. The histopathologic of the product of the nasal resection revealed persistence of the tumor on the side surgical margins.

## DISCUSSION

Sweat gland carcinoma is an unusual skin cancer that has no uniform classification, characteristic and behavior. The most common type is the EC, but it represents less than 0.01% of all skin tumors.^1,3,4^ There are multiple types of EC, such as ductal EC, eccrine porocarcinoma, mucinous EC, clear cell eccrine hidradenocarcinoma, adenoid cystic EC, digital papillary EC, microcystic adnexal carcinoma, eccrine spiroadenocarcinoma, malignant mixed tumor and mucoepidermoid carcinoma.^5^

Ductal EC is the most common, and among its histologic variants are: a) ductal EC with abundant fibromyxoid stroma; b) ductal EC with fusiform cells and myoepithelial differentiation; c) basaloid cells carcinoma; and d) SEDC, characterized by squamous metaplasia.^3^

The latter variant is extremely rare. Typically it presents as a solitary dermal nodule, ulcerated or not, in the head, neck, extremities or trunk of middle-aged or elderly individuals.^1,2^ There are reports of lesions with evolution of months to 10 years before the initial biopsy. The largest reported tumor, so far, was 27 mm in diameter.^3^

**Figure 6:**
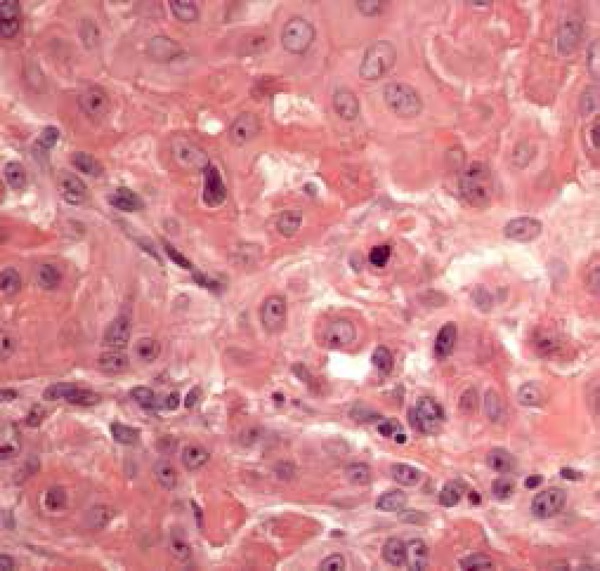
Squamoid area of cancer (HE, 400x)

**Figure 7:**
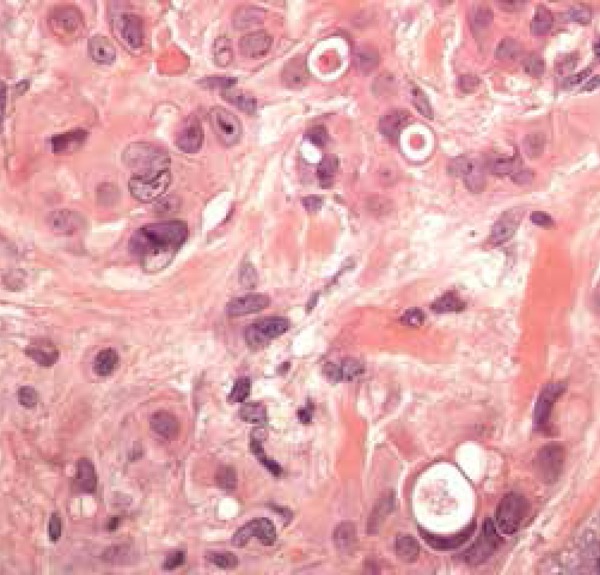
Area with ductal differentiation (HE, 400x)

Histologically it has an infiltrative and poorly delimited growth pattern, extending deep to the dermis and hypodermis. There is prominent squamous differentiation, more apparent in the upper region, where the neoplastic aggregates are larger and composed of epithelial cells with abundant cytoplasm amphiphile (Figure 6).^1,3^ Thus, when superficial biopsies are performed, the chances of incorrect initial diagnosis of SCC increase, as can be seen in almost half of published cases.^3^ In the central and deep areas of the tumor, the neoplastic aggregates are basaloid, angulated, and display tubular structures that resemble a benign syringoma (Figure 7). Atypical pleomorphic cells and mitotisis are present.^3^

The differential diagnosis includes SCC, metastatic carcinoma with squamous features and other adnexal eccrine carcinoma, such as microcystic carcinoma and porocarcinoma with squamous differentiation.^2^ The EC immunohistochemical profile comprises positivity for S-100 protein, EMA, cytokeratin and CEA. In our case, there was positivity for EMA and CEA, which are typical of glandular tissue. CK7 also marks this tissue. Combination of p63 and cytokeratin 5/6 is useful for differentiating primary cutaneous malignant disease, in which they are positive for metastatic disease.^1^

The diagnosis of this cancer is challenging because of its rarity and superficiality of many biopsies.^3^ Differentiation between SEDC and other diseases is important for the proper managing of the case, since it has a more aggressive local behavior, with a pattern of deep infiltrative growth, perineural and intravascular invasion and potential for recurrence,^3,5,6^ characteristic clearly demonstrated in this case. Up to 50% of ECs generate metastasis, while only 0.5% of SCCs do so.^5^

Limited information on the treatment of SEDC occurs because of its rarity, however, the treatment of choice appears to be a wide surgical excision with clear margins (whether or not using the Mohs technique).^4,5^ After two resections revealing compromised margins, our patient underwent radiotherapy with curative intention. Wang, Handorf, Wu, Liu, Perlis, Galloway *et al.* in a recent review on surgery and adjuvant radiotherapy applied in high risk carcinomas of the head and neck, as in this case, obtained excellent locoregional control with acceptable toxicity.^7^ In our case, five months after the end of radiotherapy, the lesion reappeared. Due to the unavailability of surgery with Mohs technique, tumor excision was performed with biopsy by intraoperative frozen section, which revealed free margins, not corroborated by histopathology of the resected product, proving the more aggressive nature of this type of lesion.

Frouin, Vignon-Pennamen, Balme, Cavelier-Balloy, Zimmermann, Ortonne *et al*. conducted an anatomic clinical study of 30 cases of microcystic adnexal carcinoma, syringomatous carcinoma and SEDC. They concluded that there were arguments for individualization of the latter entity due to its eccrine origin, its more aggressive behavior and the possibility of its occurrence in transplanted organ.^8^ The evolution of published cases can be found in Table 1.^1,3-6,9-13^

So the SEDC is a rare neoplasm, difficult to diagnose in the initial biopsy, especially if it is superficial. Its distinction from SCC is important because of its aggressive nature and the need for surgical treatment with wide margins to avoid recurrence of lesion.
